# Insights on leveraging virtual reality as a medium for intervention delivery for older adults

**DOI:** 10.1093/geroni/igaf122.3796

**Published:** 2025-12-31

**Authors:** George Mois, Emre Eraslan, Tongxin Sun, Qiyuan Cheng, Avinash Gupta, Wendy Rogers

**Affiliations:** University of Georgia, Athens, Georgia, United States; University of Illinois Urbana-Champaign, Champaign, Illinois, United States; Tsinghua University, Beijing, Beijing, China (People’s Republic); University of Illinois Urbana-Champaign, Champaign, Illinois, United States; University of Illinois Urbana-Champaign, Champaign, Illinois, United States; University of Illinois Urbana-Champaign, Champaign, Illinois, United States

## Abstract

Virtual reality (VR) is a growing immersive technology with potential to enhance access and adaptability to sustain participation in social, cognitive, and activity engagements that cannot easily be facilitated through other information communication technologies. Leveraging VR presents a unique opportunity to support resource development and improve access to services and activities that are tailored to meet older adults’ needs and preferences. However, there is limited understanding pertaining to the design and adaptation of off-the-shelf VR solutions to support adoption and use by older adults, and how VR can be used to enhance access to interventions, services, and programs. We conducted a mixed methods study in a home simulation environment to explore the application of VR as a vehicle for intervention delivery. We recruited 21 community dwelling older adults aged 61-80 (M = 69, SD = 5.45) to participate in passive VR demonstrations and gathered insights into their experience through questionnaires and a semi structured interview. Participants perceived VR as an immersive medium with the potential of adapting to their own interests and preferred activities. Our findings provide insights into design and implementation considerations in utilizing VR as a medium for intervention delivery for older adults. Based on these findings, we outline opportunities (visual and audio capabilities, tailored experiences) and challenges (disorientation, physical discomfort, usability limitations) in leveraging immersive technologies to support various types of VR engagement activities (e.g., activity, social, cognitive). These considerations can improve effectiveness of VR-based interventions and provide steps to meet the needs and preferences of older adult VR users.

